# Adapting family-based therapy for anorexia nervosa to an inpatient setting: staff experiences and perspectives

**DOI:** 10.1186/s40337-026-01706-7

**Published:** 2026-07-09

**Authors:** Jan-Vegard Nilsen

**Affiliations:** Regional Department for Eating Disorders, University Hospital in Oslo, Oslo, Norway

**Keywords:** Inpatient treatment, Family-based therapy, Anorexia nervosa, Reflexive thematic analysis, Staff perspectives

## Abstract

**Background:**

Family-based therapy is an outpatient treatment model strongly recommended as a first-line intervention for adolescents with anorexia nervosa. A central feature of the model is the active involvement of parents in meal support and in the treatment process as a whole. Although outpatient Family-based therapy is effective for many families, a substantial proportion of young people require inpatient treatment. While several specialized inpatient units have adopted core principles from outpatient Family-based therapy, empirical knowledge about how these adaptations are experienced by inpatient staff remains limited. The aim of the present study was therefore to explore inpatient staff’s experiences of working in accordance with core principles of family-based therapy within a specialized inpatient setting.

**Methods:**

The study employed an exploratory qualitative design. Data consisted of two focus group interviews with fourteen healthcare staff from a specialized inpatient unit and were analyzed using reflexive thematic analysis.

**Results:**

An overarching theme was generated: The core principles function as a dynamic compass in clinical work. Three main themes were generated: (1) Unifying around a shared professional language provides multiple advantages; (2) The treatment model increases awareness of the helper’s responsibility and therapeutic position; and (3) Adaptations are always necessary in an inpatient context.

**Conclusions:**

From the perspective of inpatient staff, the core principles of outpatient family-based therapy can be meaningfully adapted to an inpatient setting. The model appears to function as a guiding and flexible framework for therapeutic work, supporting professional coherence while allowing for necessary local adaptations. These findings highlight the potential value of principle-driven, family-based approaches within inpatient treatment for adolescents with anorexia nervosa.

## Abstract (plain language summary)

Family-based therapy (FBT) is recognized as an effective treatment for adolescents with anorexia nervosa. FBT is developed for an outpatient setting and a key part of FBT is that parents are actively involved in helping their child recover. As some young people become so unwell that they need hospitalization, treatment facilities are working to adapt the evidence-based core principles of outpatient FBT to a hospital setting. Still, little is known about how this way of working is experienced by inpatient staff. In this study, we explored how all together 14 healthcare staff experience working in line with the core principles from outpatient FBT within a specialized inpatient unit for adolescents with anorexia nervosa. We conducted two focus group interviews with staff members from different professional backgrounds and analyzed their experiences using a qualitative research method. Overall, the findings suggest that the core principles of outpatient FBT can be meaningfully and flexibly adapted for inpatient care. A principle-driven, family-based approach may support collaborative treatment for adolescents with anorexia nervosa and their families, even in highly intensive care settings.

## Background

When children and adolescents develop the eating disorder (ED) anorexia nervosa (AN), outpatient family-based therapy (FBT) is recommended as the first-line treatment [1]. FBT is a manualized form of family therapy developed specifically for the treatment of AN [2]. It can be described as an integrative model that combines psychoeducation with therapeutic stances and interventions drawn from several established family therapy traditions [3].

Although many families receive sufficient help from FBT at their local child and adolescent mental health outpatient services (CAMHS), a proportion will require more intensive support that exceeds the institutional framework of outpatient care. Outpatient FBT can be intensified in several ways. The literature suggests strategies such as adjunctive multi-family therapy [4], family-based day treatment [5], or family-based inpatient treatment [6, 7]. A common feature of these intensified treatment formats is that the core principles of the outpatient FBT model [8] are sought adapted to a more intensive treatment setting [5, 7, 9, 10].

The core principles of FBT represent a set of interrelated foundational assumptions that resonate with one another [3]. While they are described somewhat differently across the literature, they typically include: a consistent *symptom focus* throughout treatment, an *agnostic stance* toward the causes of the ED, the use of *externalizing language* when referring to the ED, a non-directive or *consultative therapeutic position*, and a sustained focus on *empowering parents*, meaning strengthening parents in their parental role, a role that is significantly challenged when a child develops AN [2, 8]. The final principle, empowering parents, is what most clearly distinguishes FBT from other therapeutic approaches [11]. In FBT, clinical work with the young person is largely mediated through the parents, particularly during the initial phase of treatment. This strong parental focus has led the model to be described as a parental empowerment model [12].

In addition to these core principles, which function as a guiding framework for the therapist throughout the course of treatment [8], outpatient FBT is structured into three overarching treatment phases [2]. In a prototypical course of treatment, as described in the treatment manual, a total of 20 sessions is distributed across these three phases. The phase of treatment corresponds to the severity and presentation of ED symptoms, and each phase is associated with distinct therapeutic tasks and goals [2].

Phase 1, which typically lasts the longest, focuses primarily on reversing starvation, ensuring sufficient weight gain, and minimizing and interrupting compensatory behaviors such as self-induced vomiting and excessive physical activity, if present. During this phase, parents assume full responsibility for the preparation and supervision of all meals. A central therapeutic aim throughout phase 1 is to form a strong alliance with the parents so that they regain confidence in their caregiving capacity while simultaneously developing the skills required to manage a new and demanding situation [2].

Phase 2 is a transition phase and largely involves supporting parents in gradually transferring responsibility for eating back to the young person. The overarching goal during this phase is to facilitate the adolescent’s increasing ownership of their own recovery process [2].

In phase 3, weight is expected to be stabilized at a healthy level consistent with the individual growth trajectory, and eating behavior normalized to a degree comparable to the period prior to the onset of AN. At this stage, greater attention is given to other relevant adolescent developmental issues and family relational themes. Therapeutic topics that were previously deprioritized due to the symptom-focused emphasis of phases 1 and 2 are now addressed more directly [2]. The specific content of phase 3 varies based on assessments of the needs of the adolescent and family. For some, treatment is concluded after a relatively brief period, whereas for others it is appropriate to continue therapy or refer the adolescent or the family for further intervention targeting remaining difficulties. Although research has demonstrated relatively low levels of sibling participation [13], FBT is designed to engage and involve the entire nuclear family in treatment [2].

Within the ED literature, the evidence base for outpatient treatment settings is relatively clear. Clinical practice guidelines consistently recommend that children and adolescents with EDs, together with their families, should first be offered outpatient FBT through local CAMHS services [1, 3]. Treatment is also recommended to be intensified if sufficient improvement is not achieved within a reasonable timeframe.

In contrast, the knowledge base for day treatment and inpatient treatment for AN is less well established. There is limited research-based evidence to guide which types of treatment intensified services should preferentially offer [14]. This is reflected in substantial variation across intensive treatment units regarding content, structure, format, and preferred therapeutic orientation. For example, a knowledge synthesis that aggregated findings from a wide range of inpatient treatment settings for children and adolescents found that only three of the sixty-six included original studies demonstrated a clearly FBT treatment profile [15]. Most inpatient ED units were characterized by an integrative or mixed theoretical approach, incorporating elements from multiple therapeutic models. The situation appears different for day treatment programs, where a considerably larger proportion are organized around a clearly defined FBT-informed framework [5].

In a Norwegian context, a large naturalistic follow-up study examined family-based inpatient treatment in a specialized inpatient unit [7]. The authors concluded that family-based inpatient treatment, drawing direct inspiration from outpatient FBT, represents a promising approach when children and adolescents require specialized inpatient care. At the same time, the study emphasized the necessity of adapting FBT to the unique characteristics of the inpatient setting and highlighted the need for further research [7, 14].

A critical source of knowledge that is currently entirely lacking, both nationally and internationally, concerns how adaptations of outpatient FBT within an inpatient framework are experienced by the staff working within these units. To date, existing studies have focused on ED outcomes at follow-up [6, 7, 16] and exploring family members’ perspectives on treatment [17, 18]. Thus, no scientific studies have examined how staff experience working with a family-based approach to AN in an inpatient setting. This represents a significant gap in the literature, given that staff are responsible for implementing and sustaining a treatment model in everyday clinical practice. Developing knowledge about staff experiences of a particular treatment approach is essential for understanding the meaning they attribute to the model and for informing ongoing clinical development and research. Without systematic investigation, such knowledge remains anecdotal and locally bound, and therefore inaccessible to others. This gap in the evidence base provides the foundation for the present study. The research question was formulated broadly as follows: *How do staff experience working in an inpatient setting inspired by outpatient family-based therapy?*

## Method

### Research design

The study employed a qualitative, exploratory design using reflexive thematic analysis (RTA) [19, 20] to examine staff experiences of adapting principles of FBT within an inpatient setting. The empirical data consisted of two focus group interviews conducted with clinical staff at a specialized eating disorder unit (EDU).

### Study context and data collection

#### The inpatient treatment setting

The study was conducted at a specialized inpatient EDU operating within a family-based treatment framework. Since 2008, the unit has provided family admissions rather than individual admissions [7]. In this model, where 6–8 families may be admitted simultaneously, parental presence during admission is flexible and varies according to practical and clinical considerations. Parents typically alternate their presence on the ward, for example, by attending on alternating days or weeks, depending on family circumstances and what is considered therapeutically appropriate. In most cases, at least one parent is present with the adolescent for substantial parts of the admission, and, when feasible, both parents are encouraged to participate during key phases of treatment. Although the ideal is for the whole family to be present, this is often difficult to sustain over time due to practical constraints [21].

The central goal of treatment is to support and strengthen parental capacity to respond effectively to both the ED and the adolescent’s emotional needs. In line with core principles of FBT, parents are supported in taking an active and leading role in treatment, and they retain primary responsibility and the final say in many aspects of care within the therapeutic framework. Siblings may also be actively involved in treatment when possible. The treatment team places emphasis on attending to siblings’ roles, experiences, and well-being, and siblings are actively invited into the therapeutic process. This may include participation in family therapy sessions, involvement in selected meals, opportunities to meet other siblings in similar situations, and access to individual supportive conversations with clinicians [14].

The duration of admission varies depending on clinical need, but treatment is generally time-limited and oriented toward supporting transition to outpatient care. A central aim is not prolonged inpatient stabilization, but to support families in developing sufficient confidence and competence to manage the ED at home [7]. The inpatient context differs in important ways from standard outpatient FBT. While outpatient FBT emphasizes parental responsibility with limited direct intervention from clinicians, inpatient care necessarily involves greater staff involvement due to the need for medical monitoring, risk management, and maintaining the structure of care. Staff therefore take an active role in ensuring safety and daily routines, while simultaneously working to support and gradually transfer responsibility to parents. As such, treatment involves an ongoing negotiation between ensuring clinical and structural safety within the inpatient setting and maintaining a therapeutic stance that promotes parental autonomy and authority. Adaptations of FBT within this context include more directive staff involvement in high-risk situations, structured meal support within the ward environment, and the use of the inpatient setting as a context in which parents can practice and consolidate caregiving skills under guidance [14].

This approach may be best understood as a hybrid model, in which core principles of outpatient FBT, such as externalization of the illness, prioritization of weight restoration, and a strong focus on parental empowerment, are retained while being adapted to the relational and organizational demands of inpatient care. Compared to many inpatient programs internationally, where family involvement may be more limited or variable [15], the present model places sustained emphasis on parental presence and participation throughout the admission.

In this regard, the model also shares important similarities with FBT-informed day treatment programs [5] or partial hospitalization programs (PHP) [22, 23], where family involvement and parental responsibility are central. The primary distinction lies in the inpatient setting’s provision of continuous, 24-hour care and the associated responsibility for medical monitoring and risk management. However, the underlying therapeutic focus, values, and clinical stance, particularly the emphasis on parental empowerment and active family involvement, are broadly consistent across these levels of care.

#### Data collection

Data collection took place at two time points: the first focus group was conducted in March 2020, at the outset of the training program (see below for further details), and the second toward the end of the same year, following program completion. The interviews lasted approximately 60 and 90 min, respectively. Although data collection took place in 2020, during the COVID-19 pandemic, at the time of data collection, the pandemic did not significantly affect staffing, the implementation of the training program, or the conduct of the focus groups. The group format was chosen to facilitate discussion of shared clinical experiences and collective reflections on practice. However, it may also have influenced the data through group dynamics, including potential social desirability and conformity pressures. The focus groups were conducted in Norwegian, and the interviews were transcribed verbatim in the original language. Quotations presented in the manuscript were subsequently translated into English by the author for the purposes of reporting.

Both focus groups were co-moderated by the author and a colleague with clinical experience in the unit. The author is a clinical psychologist, PhD, and family therapist with approximately 20 years of experience working in adolescent EDs, including extensive clinical experience in higher levels of care. The co-moderator is a clinical psychologist and family therapist with more than 30 years of experience in the field of adolescent EDs, including inpatient treatment. She also holds a university degree in philosophy. Both moderators have substantial experience in teaching standard outpatient FBT, supervising clinicians and students, and working within FBT-informed outpatient settings.

#### The training program

The focus group interviews were conducted in connection with an internal staff training program delivered at the EDU in 2020. The program was primarily based on an established outpatient FBT training program that the EDU had offered over several years to professionals working in outpatient settings within its tertiary catchment area. The original program comprised five full days of lectures and five days of group-based supervision. This model was subsequently adapted for the inpatient context. The adapted program consisted of four full training days, followed by five supervision sessions of three hours each, amounting to a total of 15 h of supervision. Staff were divided into three supervision groups. While the four full training days retained the core content and therapeutic focus of the original outpatient FBT program, the emphasis of lectures, group tasks, role-play exercises, and reflective discussions was adjusted to address clinical dilemmas and therapeutic situations more commonly encountered in inpatient care.

Another important contextual factor is that the inpatient EDU in which the study is situated has operated within an overarching family-based inpatient treatment framework since 2008 [7]. Accordingly, the training program did not introduce a new treatment model; rather, it marked the first occasion on which the EDU conducted a more systematic, unit-wide training initiative explicitly informed by outpatient FBT. The author was not involved in the development or delivery of the training program. The training was conducted by two senior clinicians, each with more than thirty years of experience in clinical practice and teaching, who were not working at the EDU. Although the training program, named “FBT adapted to an inpatient setting”, was conducted in 2020, the EDU has not formally changed its overarching family-based inpatient treatment model since that time. The program therefore reflects an approach that remains broadly consistent with current clinical practice at the EDU. While the model has remained formally unchanged, local adaptations over time cannot be ruled out.

### Participants

Twenty-one staff members participated in the training program, of whom fourteen took part in the focus groups. Three participants attended both focus groups; thus, most participated in only one of the two groups. All staff members who took part in the training program were invited to participate in the focus groups. Participants primarily consisted of clinical milieu staff working in the inpatient unit, the majority of whom were registered nurses. Some participants had additional training or further clinical qualifications, including two family therapists. The sample also included one clinical psychologist and one psychiatrist. Detailed demographic and professional background data were not systematically collected. However, participants represented a multidisciplinary clinical team with varying levels of experience and training relevant to the treatment of EDs. As such, potential differences in perspectives across professional roles and levels of training could not be systematically explored in the analysis. Participants had between 2 and 12 years of experience working in higher levels of care.

As most staff worked shifts within the clinical milieu, the focus groups were scheduled around handover periods, when day staff exchanged with afternoon staff. Both focus groups were arranged by the ward manager. All but one participant were women. Detailed information about non-participants was not systematically collected. Given that participants were recruited from shift-based clinical staff, non-participation was likely primarily due to practical constraints related to scheduling and availability.

### Interview guide

Prior to the interviews, the author and the colleague co-moderating the focus groups developed an interview guide, which served as the basis for both focus group interviews. The guide functioned as a flexible point of departure; the questions were posed to the group as a whole and were intended to facilitate discussion and the sharing of experiences among participants. The suggested questions included: “How have your experiences been so far with participating in the training program – FBT adapted to an inpatient unit?”, “Which of the core principles of outpatient FBT do you find most relevant or important in your work?”, “Has what you have learned so far influenced your previous clinical practice, and if so, how?”, “Can you provide examples of how the central principles of FBT shape your thinking and actions in different clinical situations?”, “How do you notice changes in your practice, and how do you think these changes are perceived by others?”, and “Do you experience any barriers to applying your newly acquired knowledge, and can you provide examples?”.

### Analysis

Braun and Clarke’s pragmatic framework for thematic analysis (TA) [24] was used to develop the findings. This involved following the six phases associated with Braun and Clarke’s approach to TA, now referred to as RTA [24, 25]. In practice, the framework functioned as a guiding tool in the detailed analytic process rather than as a strictly linear procedure to be followed step by step [26]. The analysis was primarily data-driven, aiming to capture participants’ experiences, while acknowledging the interpretative and constructive nature of theme development [25, 26]. Coding was conducted manually by the author as part of the iterative analytic process. The process of coding data was also aligning with the preferred stance embedded in RTA, as “… open and organic, with no use of any coding framework” [26, p. 334].

#### Reflexivity

In line with the principles of RTA, the researcher’s subjectivity was considered an integral part of the knowledge production process. As mentioned above, the author is an experienced clinical psychologist, PhD, and family therapist. Importantly, the author previously worked at the study site between 2006 and 2010, and again between 2014 and 2016. However, none of the participants in the present study were employed at the EDU during that period. As such, the author did not have direct collegial relationships with participants at the time of data collection. Nevertheless, familiarity with the clinical context may have influenced both data collection and interpretation. Informed by Braun and Clarke’s increased emphasis on researcher subjectivity [19, 20, 26] and by the recognition that qualitative analysis is inherently a constructive and creative process, the author kept reflexive notes throughout the analytic process. These notes were used to document reflections, perceived dilemmas, and subjective perspectives related to the development of the findings.

Several particularly relevant and recurring critical aspects of the study were identified. First, the author conducted the analysis independently, without systematic discussion of the emerging findings with other researchers. Second, a central and potentially critical issue is that the author conducted research in his former workplace. When research is situated in a context in which the researcher has invested several professional years, it becomes especially important to remain attentive to how such contextual factors may influence the analysis [14]. This concern is further intensified when the researcher conducts the analysis alone and is solely responsible for conveying the narrative derived from the data. A key strategy for addressing this challenge involved repeatedly returning to the raw data to ensure that theme development was adequately grounded in participants’ accounts. A continuous aim was thus to reduce the risk of constructing a narrative of staff experiences that primarily reflected the author’s own preferences and theoretical orientations rather than the empirical material.

### Ethics

The study was approved by the Data Protection Officer at Oslo University Hospital, and all data were stored in accordance with institutional requirements for data security and confidentiality. All participants provided written informed consent at the start of the study, and participation was voluntary. It is not possible to identify individuals from the illustrative quotations presented in the results section.

## Results

The analysis generated one overarching theme and three main themes reflecting shared patterns across participants’ accounts. The overarching theme, “The core principles function as a dynamic compass in clinical work,” encompasses three main themes.

Main Theme 1: “Unifying around a shared professional language provides multiple advantages.”

Main Theme 2: “ The treatment model increases awareness of the helper’s responsibility and therapeutic position.”

Main Theme 3: *“*Adaptations are always necessary in an inpatient context.*”*

Selected quotations are presented both immediately following certain theme headings and throughout the text to provide examples from the raw data and to illustrate key analytic points.

Overarching theme: The core principles function as a dynamic compass in clinical work

…it is extremely useful for us to be as aligned as possible; it is important that we share a common framework grounded in theory….

Participants emphasized that working with severe AN in an inpatient setting, where several families are admitted simultaneously, represents a highly complex and demanding clinical context. Across the discussions, it became clear that this complexity heightened the perceived need for a shared professional framework around which staff could align and from which treatment could be flexibly adapted. Overall, participants in both focus groups largely agreed that the core principles of the FBT model, despite having originated in a markedly different clinical context, provide substantial support when navigating the complexities of inpatient care. In this sense, these principles were understood as functioning as a dynamic compass for staff, rather than “…a new straitjacket we have to wear.” Staff perspectives on the FBT model were thus characterized as dynamic, in that the framework allowed sufficient room for necessary local adaptations, and as a compass, in that it offered clear professional direction for therapeutic work. As one participant reflected:

“… it’s not a manual where you just follow a recipe… it’s more like a framework… and then we have to figure out together how to make it fit our context…”.

Main Theme 1: Unifying around a shared professional language provides multiple advantages

…we may be doing much of the same work as before, but now we have a clearer professional language for what we do….

When participants talked about the FBT model, it became evident that they primarily experienced the model as providing a language, comprised of professionally meaningful metaphors and concepts grounded in family therapy theory. As one participant reflected: “… we get a shared language for what we’re doing… and that makes it easier to understand both each other and the families…” Several participants referred to this terminology as “the FBT language” or “FBT jargon.” Participants also appeared to agree that this “FBT language” represents a form of rhetoric that resonates with their concrete clinical experiences in the inpatient unit. In this way, it is a language that is experienced as meaningful.

All of the core principles as described in the professional literature were mentioned as useful “guidelines” or premises for clinical work at the unit. Among these principles, particular emphasis was placed on the primary task of strengthening parents in their parental role during the admission, illustrated using the English expression “empowering the parents”, as well as the importance of maintaining “the consultative therapeutic position.” These were explicitly highlighted as particularly helpful metaphors.

Being unified around such a shared “FBT language,” as participants described it, appeared to make several differences in staff members’ experiences of their work. One such difference concerned improved communication and collaboration, both internally and externally. Internally, the “FBT language” was described as useful in discussions within the staff group and in the many concrete dialogues staff have on a daily basis with family members during their admission. Externally, participants referred to advantages associated with having access to a shared language in conversations with collaborating services before, during, and after inpatient admissions. As another participant described: “… it’s not just internally… but also when we talk to outpatient services… we can recognize the same concepts and know where they’re coming from…”.

With access to a shared professional language, communication was thus perceived to flow more smoothly, “… when everyone is more or less speaking the same language,” both within the EDU and in contact with referring services and other collaborators. The language therefore seemed to function not merely as a local internal jargon, but as part of a shared vocabulary that extended beyond the inpatient stay and was recognizable both prior to and following admission.

Subtheme: Regular supervision should be ongoing to strengthen practice.

Although the inpatient unit has worked within a family-based framework for many years, several participants emphasized the necessity of continuous professional development. This was partly attributed to staff turnover, ”… staff come and go”, and partly to the risk that factors may emerge over time that challenge the preferred clinical approach, leading to a gradual dilution of the treatment model and the associated “FBT language.” Participants were unanimous in expressing that while training and supervision are experienced as valuable in the short term, education, and supervision in particular, must be sustained over time. The need for supervision does not diminish: “… we need continuous supervision, maybe not three hours… [as in the training program] … but ideally weekly or every other week.”

Having the entire staff group participate in a tailored training program with supervision was highlighted as making a positive difference. Participants described a strengthened sense of shared purpose and collegial cohesion. Several noted that although they had received supervision previously, this format differed from earlier experiences because it was more explicitly grounded in theory and oriented toward integrating clinical practice with theoretical principles, rather than primarily focusing on sharing experiences within the group. As a result, supervision was experienced as more purposeful and more clearly anchored in the treatment model. As one participant noted: “… it forces us to reflect in a more structured, professional way… not just sharing frustrations, but actually working through the cases…”.

Main Theme 2: The treatment model increases awareness of the helper’s responsibility and therapeutic position.

Across both focus groups, recurring reflections emerged regarding how staff understood their responsibility toward both the parents and the adolescent with AN. Participants largely positioned themselves within a helper or therapeutic role that resonated with the explicit parental focus of the outpatient FBT model. As one participant described: “… we always try to support the parents… so they can take responsibility… rather than us stepping in and taking over…”.

This therapeutic position, where the alliance with the parents is consciously prioritized, was described by one participant using a metaphor translated as a “flyer function,” referring to a way of working in which clinicians move (i.e. “fly”) flexibly in and out of challenging situations. Rather than taking over parental responsibility during difficult mealtimes, the clinician “steps in” to support the parents through collaborative problem-solving, and then actively withdraws to allow the parents to manage the situation themselves, thereby reinforcing parental authority and competence. When this metaphor was introduced, “… like a flyer”, it was evident that the concept resonated strongly across the group, suggesting that it captured a shared understanding of the helper’s role and positioning within a family-based inpatient framework. This metaphor reflects a core tension in the inpatient adaptation of FBT, where clinicians must remain actively involved within a highly structured setting while simultaneously resisting the impulse to assume parental responsibility. Another participant mirrored this stance while reflecting on staff availability:

…we are so available here… we can easily take up too much space… but with the FBT thinking in mind, we have to pull back… and let the parents handle it… even if we could step in….

Subtheme: Facilitating parents’ decision-making capacities

… our task is to ‘empower the parents,’ not to make the child well ourselves….

A recurring central therapeutic task concerned the importance of facilitating situations within the EDU in which parents are given genuine opportunities to make and take ownership of decisions, and, importantly, to develop greater capacity to stand firm against the many demands and imperatives imposed by the ED. This approach stands in contrast to practices in which the treatment team or individual clinicians make decisions on behalf of the family and subsequently act as intermediaries who convey those decisions.

Several participants acknowledged that it can nevertheless be “… very tempting to try to rescue the situation,” for example by providing more direct emotional support to the adolescent during a particularly challenging meal, or by working more directly with the young person in similarly difficult situations. The risk that parents may then be relegated to the background as passive observers was highlighted as a significant dilemma in such contexts. Although examples of working more one-to-one with the adolescent were not described as incorrect practice, the articulated ideal consistently emphasized working “through the parents first.” As one participant reflected: “… it’s so easy to turn to the young person… that’s the natural thing to do… but actually staying with the parents takes more effort…”.

Actively consulting parents to a greater extent than before was evident in participants’ reflections on how decision-making processes were approached in weekly treatment meetings. There was consensus that parents should be consulted first, prior to establishing or modifying treatment agreements. A clear parental focus was also apparent in descriptions of planning leave from the unit, as well as in the many more spontaneous situations that arise in inpatient care: “… we always try to consult the parents first… even though it’s not always easy…” In this context, periods of leave were described as “self-efficacy training” and several participants emphasized how preparatory planning before leave should contribute to increasing parents’ awareness of the support and assistance they need in order to manage potentially difficult situations at home. This shift suggests that empowering parents is not only understood as a treatment goal, but as an active clinical process embedded in everyday decision-making and interactions.

Main Theme 3: Adaptations are always necessary in an inpatient context

“…it’s not ‘one size fits all’… not everything from outpatient FBT can be transferred directly here… we have to figure out how to make it work in this context…”.

The theme of adaptation reflects both participants’ more general perspectives on the importance of tailoring treatment to the individual adolescent and family, because “… people are different”, and their more specific perspectives and experiences regarding how outpatient FBT can be adapted to, or fit within, an inpatient setting. Reflections on adaptation often stemmed from reminders about the unique nature of specialized inpatient treatment and its position within the broader continuum of care. As one participant stated, “… they’re not supposed to stay here with us until treatment is finished…”.

Several participants also reminded one another that adolescents and families admitted to the EDU have typically already attempted various family-based interventions in their local services. As one participant noted: “… these are often complex cases… they’ve usually tried FBT for a long time already… so we have to think more broadly… what hasn’t been tried yet…” Some may also have experienced one or more admission within local specialist healthcare services. In such cases, both families and treatment teams may begin to question whether continued family-based treatment remains the most appropriate approach. As one participant reflected, “[standard outpatient] … FBT works for maybe 70% at best, or something like that … we also need to offer something to the remaining 30% …”.

Staff appeared largely unified in their belief that within the inpatient context, and still operating within a family-based framework, “… we have to offer something different,” rather than repeating approaches that have not been effective thus far.

Subtheme 1: Intensifying work with family meals.

Providing mealtime support was regarded as one of the most important therapeutic tasks during an inpatient admission, perhaps the most important of all. As one participant stated, “… that’s really why they’re… um… here for, because they’re not managing meals at home.” Consequently, several participants reflected on how work with family meals is adapted during an inpatient admission.

In outpatient FBT, meals constitute a highly relevant topic of therapeutic discussion; however, the therapist is less directly involved in meals as they don’t occur physically in the therapy room. In the inpatient unit, work with meals is considerably intensified. There is potentially even more discussion about meals, about what interferes with them and how parents can best provide support, and there is also far more intensive in vivo work with meals themselves.

Another important difference is that meals during an admission tend to be more regulated than what is considered ideal within outpatient FBT. In the outpatient model, parents are largely expected to determine how individual meals are structured and carried out. As several participants noted, in an inpatient unit both physicians and dietitians are often involved in the multidisciplinary team. Although efforts are made to facilitate active collaboration with parents in these processes, the inpatient unit also imposes certain non-negotiable frameworks. For example, it is not possible to bring personal food into the unit, and dietary plans must generally be increased if the adolescent does not gain sufficient weight, regardless of parental opinions on the matter.

These structural conditions may challenge the consultative and non-directive therapeutic position that characterizes outpatient FBT. One participant expressed this tension as follows:

“… some of the structures we’ve created here… they help reduce conflict, but they can also become a kind of crutch… and then maybe make things more difficult when the family goes home…”.

This highlights a central dilemma in the inpatient adaptation of FBT: structures that support families during admission may simultaneously risk undermining their confidence when responsibility is returned to them at home. Nevertheless, against the backdrop of these dilemmas, participants highlighted the potential inherent in intensive work with family meals within the inpatient setting as a significant opportunity to support families’ progress. As one participant remarked, prompting several nods of agreement from the group, “It’s quite fascinating that in standard FBT they have one single meal session… um… and we might have four a day, right?” Thus, in the inpatient context, work with family meals takes place actively “… throughout the entire day, the entire week, the entire admission.”

Subtheme 2: The phase model must be adapted to the unique inpatient setting.

Several participants repeatedly returned to the view that the “phase model,” as described in outpatient FBT, is the aspect of the model that is least directly transferable to an inpatient setting. Examples were given of how staff had, over time, developed local expressions such as “… phase zero” or even “… phase minus one.” These were described as locally developed metaphors for processes in which families, particularly parents, were assessed as “… not yet ready for the parental tasks or responsibilities associated with FBT phase 1.”

In outpatient FBT Phase 1, parents are expected to manage all meals themselves, with support from the therapist. At the start of an inpatient admission, however, it may have been months since some parents last had primary responsibility for meals. Another important adaptation of the phase model concerned the perceived necessity of working therapeutically with issues and themes associated with all three phases simultaneously during an inpatient admission. As one participant emphasized, “… these families, and the young person especially, really need to be able to focus on completely different problems than weight or food… that may be what they need more than anything right now… to be stimulated and re-engaged with life outside again…”.

Whereas in outpatient FBT phase 1 the therapist deliberately postpones addressing therapeutic themes other than nutrition, meals, weight, and compensatory behaviors until ED symptoms have subsided, several participants made it clear that during an inpatient stay staff must allow for greater thematic flexibility, “… regardless of where the patient is positioned,” according to an outpatient phase model. As one participant stated emphatically: “… we quite simply cannot be literally focused on food 24/7…”, and another participant added: “… we try things out… we don’t always know what will work… we try and then we adjust…” This reflects a shared understanding of treatment as an iterative and context-sensitive process, rather than a linear progression through predefined phases.

## Discussion

This study extends the FBT literature by providing an in-depth account of how inpatient staff experience adapting outpatient FBT principles to a specialized inpatient setting. Staff perspectives on a unit’s treatment approach and their own clinical work represent an important source of knowledge. Alongside research on ED outcomes [6, 7, 16], treatment satisfaction [27], and studies examining family members’ subjective experiences of care [14], a deeper understanding of how staff perceive a unit’s preferred clinical practice may contribute to further development of treatment provision for those requiring inpatient care. Examining staff perspectives is particularly relevant for understanding whether clinicians’ experiences and viewpoints resonate with an organization’s preferred treatment approach, in this case, family-based inpatient treatment for AN [7].

Overall, the findings indicate that staff recognize that the core principles characterizing outpatient FBT can be transferred to an inpatient setting. In this way, the study supports existing research suggesting that despite the evident differences between outpatient and inpatient contexts [9], the core principles of FBT [8] can be adapted to an inpatient treatment framework [6, 7, 28]. The results further demonstrate that staff perceive several advantages in drawing inspiration from a specific therapeutic framework, such as FBT [2], in inpatient clinical work. Notably, the findings suggest that the core principles are primarily viewed as a flexible and guiding framework for practice, one that both provides direction for treatment and allows for necessary adaptations [29].

These findings resonate with the broader psychotherapy research literature, which over several decades has demonstrated that although it is nearly impossible to establish the superiority of one particular therapeutic model over another, it remains crucial that therapists are educated and trained within a coherent therapeutic framework [30–32]. Offering treatment within a single model or framework that provides both therapist and patient with a coherent understanding of how psychological difficulties emerge, are maintained, and can be changed appears essential [30, 31]. This also seems to hold true for staff working within an inpatient context.

One way of conceptualizing differences between therapeutic approaches is to view them as offering therapists different professional languages, both for understanding clinical phenomena and for guiding therapeutic action in interactions with patients and families. Such linguistic dimensions were clearly highlighted in Main Theme 1, where the FBT model was experienced as providing a shared professional language among staff. This is a language actively used in everyday clinical work at the EDU, grounded in theory and supported by decades of empirical research [2, 33]. It is reasonable to assume that this shared language not only facilitates smoother communication both internally and externally, as indicated by the findings, but also provides staff with a sense of professional legitimacy and authority.

The FBT language, with its distinctive metaphors reflected in the core principles [8], may also contribute to increased clarity regarding the central therapeutic tasks during an inpatient admission. Put simply, the overarching goal of a family admission is to support the family in reaching a point where they are able to manage sufficiently with continued support at a lower level of care [7, 34]. Achieving this requires treatment to adequately equip parents to manage the ED at home, outside the protective structure of the inpatient EDU.

Awareness of responsibility, therapeutic positioning, and the importance of facilitating strengthened parental competencies during an admission were central to Main Theme 2. The non-directive or consultative therapeutic stance that characterizes outpatient FBT [2, 8] may be particularly challenged within the highly structured medical environment that inpatient units typically represent [10]. The literature has called for staff to demonstrate creativity and actively promote parental authority and competence in contexts where clinicians traditionally make many decisions on behalf of the admitted adolescent and family [9, 10, 18]. The present findings indicate that these relational dynamics are issues staff continually attend to and work to develop. This is important, as some parents may experience remaining on the sidelines even during a family-based admission. Despite treatment efforts aimed at strengthening parental involvement, some parents may continue to experience a sense of powerlessness in relation to the ED and their child [18]. The findings provide several examples of staff actively striving to remain attentive to such dynamics. That staff consistently seek to create situations in which parents can practice reclaiming a parental position that the ED has undermined [35, 36] is a reassuring finding from a family-based perspective.

A related aspect that warrants further consideration is the interplay between parental empowerment, staff support, and the experience of demoralization among families in an inpatient context. While promoting parental empowerment is a core principle of FBT [8], families admitted to inpatient care often do so at a point where confidence, agency, and perceived competence may be significantly reduced [36]. In contrast to outpatient settings, where treatment typically builds on an initial assumption of parental capacity to take the lead [2], inpatient admission may reflect situations in which families experience themselves as overwhelmed, uncertain, or unsuccessful in managing the ED. This creates a clinical paradox: the therapeutic task remains to empower parents, yet this must be done in a context where parents may initially feel least able to assume such responsibility. The present findings suggest that staff play a crucial role in navigating this tension. By offering structured support, modelling interventions, and creating contexts for parents to gradually resume responsibility, staff may help bridge the gap between demoralization and empowerment. At the same time, there is a risk that staff involvement, particularly in a highly structured inpatient environment, may inadvertently reinforce parental passivity if not carefully managed [10, 18]. These dynamics highlight the importance of a carefully calibrated therapeutic stance, in which staff support is provided in a way that actively promotes, rather than replaces, parental agency. This balance may represent a key mechanism through which FBT principles can be meaningfully adapted to inpatient care. Although the mechanisms of change in FBT are not fully understood, strengthening parents’ self-efficacy and coping skills is widely regarded as one of the most effective elements of the model [37–40]. Consequently, for an inpatient unit to credibly describe its treatment as FBT-inspired, facilitating situations in which parents experience themselves as truly *empowered* appears essential.

Adaptations of the model to the unique clinical context in which staff operate were addressed in Main Theme 3, which also illustrated examples of locally developed adaptations of the FBT framework. There are many ways of conceptualizing and organizing inpatient treatment for EDs, a reality reflected in a systematic review of psychological outcomes following inpatient treatment for adolescents with EDs. This review highlights substantial international variation in inpatient services with respect to treatment duration, overall structure, program content, and preferred theoretical and therapeutic orientation [15].

An important issue when one seeks to inform inpatient treatment from an outpatient treatment model, as FBT [2], concerns the inherent tension between maintaining fidelity to core principles of FBT and the need for flexibility in adapting the model to the unique inpatient context. While participants consistently described the FBT framework as providing a clear therapeutic direction, they also emphasized the necessity of adapting its application to the realities of inpatient care. This tension reflects a broader dilemma in psychotherapy implementation, where strict adherence to a manualized model may be difficult to sustain across different clinical contexts, yet excessive flexibility risks diluting the defining features of the approach. In the present study, staff appeared to navigate this dilemma by orienting themselves toward the underlying principles of FBT, particularly parental empowerment, externalization of the illness, and prioritization of weight restoration, rather than rigidly adhering to specific procedural elements. The findings suggest that such a principle-driven approach may allow for meaningful adaptation without fundamentally departing from the therapeutic model. At the same time, this raises important questions regarding where the boundaries between acceptable adaptation and loss of model integrity should be drawn. This issue is particularly salient in inpatient settings, where structural and medical demands may necessitate more directive interventions than those typically associated with outpatient FBT [9, 10]. These considerations highlight the importance of ongoing supervision and reflective practice in supporting clinicians to balance fidelity and flexibility in a way that remains consistent with the underlying therapeutic intent of the model [29].

Whether inpatient treatment is grounded in a systemic framework [14], cognitive behavioral therapy [41], or an integrative approach [15] may be less important than whether staff share a coherent understanding of how to address clinical challenges. The ED field has a considerable distance to go before it can offer more evidence-based conclusions regarding whether outcomes after inpatient care differ depending on whether staff are aligned around one model versus another [15]. Overall, it is notable that the findings of this study suggest that staff experience room for flexibility and adaptation within a FBT framework, particularly given that research has struggled to establish a direct relationship between strict adherence to a specific treatment manual and treatment outcomes [29].

### Strengths and limitations

To the author’s knowledge, this is the first study to examine staff experiences of working with a family-based approach to severe EDs within an inpatient setting. The study thus addresses an underexplored area, one in which substantial human and financial resources are invested to ensure high-quality care for individuals requiring inpatient treatment. In this context, a clear strength of the study is that it provides an initial, exploration of staff perspectives and experiences of working in this way. The findings may serve to inspire other services considering a shift toward more family-based practice, as well as support continued professional development in units already working within a family-based framework.

Nevertheless, several potential limitations warrant consideration. As reflected in the methods section, the study was conducted by a single researcher who previously worked at the EDU where participants were employed. It cannot therefore be ruled out that the study in general, and the analysis in particular, may be biased toward presenting a predominantly positive narrative regarding the adaptation of outpatient FBT to an inpatient context. Although there was no explicit intention to promote or defend one practice over another, such unintended bias cannot be entirely excluded.

Importantly, the analysis was conducted by a single researcher, without the use of cross-coding or member checking. While these approaches are often associated with enhancing credibility within some qualitative traditions, RTA does not conceptualize reliability in terms of coder agreement or validation through participant verification [19, 26, 42]. Instead, the analytic process involves an interpretative engagement with the data, where the researcher’s subjectivity is acknowledged as a resource rather than a bias to be eliminated [20, 25]. However, the absence of additional analysts or participant validation may still have limited the range of interpretations considered, and the findings should therefore be understood as *one* possible, contextually situated interpretation of the data rather than a definitive representation.

Another potential limitation is that the focus group discussions may have favored favorable accounts of how staff succeeded in adapting FBT to inpatient care, rather than facilitating more in-depth discussion of dilemmas, including experiences of challenges and difficulties. The study may also be criticized for not sufficiently exploring certain clinical dilemmas, such as how staff address comorbidity, how clinical work is shaped when adolescents present with low motivation for change, how family conflict is managed, or how teams respond when a family-based approach is deemed less appropriate for a particular adolescent or family. Further research is therefore needed to develop more nuanced understandings of how staff both experience and manage complex clinical dilemmas within inpatient ED treatment.

Additionally, detailed information regarding non-participants was not systematically collected, limiting the ability to assess potential differences between participants and non-participants. While participation was likely influenced by practical constraints such as shift schedules, this raises the possibility that the data may reflect the perspectives of those more available or motivated to participate.

## Conclusions

From the perspective of the staff group included in this study, the core principles that characterize outpatient FBT can be meaningfully adapted to a specialized inpatient context. The findings suggest that these principles function as a coherent and flexible framework that provides clinical direction while allowing for necessary contextual and local adaptations. In a complex inpatient environment, where treatment demands are intensive and relational dynamics multifaceted, being united around a shared theoretical model appears to support both professional coherence and reflective clinical practice.

Importantly, staff described the family-based framework not as a rigid protocol, but as a guiding orientation that promotes consistency, shared language, and heightened awareness of therapeutic responsibility, particularly in relation to strengthening parental competence and authority. These features may be especially valuable in inpatient settings, where structural and medical demands risk displacing parents from an active caregiving role.

Overall, the findings suggest that adaptations of FBT may represent a viable and clinically meaningful approach to inpatient treatment for adolescents with AN, from the staff perspective. Further research is needed to examine how such adaptations influence clinical processes and outcomes, and to explore staff experiences across different inpatient contexts.

### Clinical implications

The findings of this study have several implications for inpatient services providing treatment for adolescents with AN. First, they suggest that inpatient units may benefit from organizing care around clearly articulated core principles derived from FBT, even when the full outpatient model cannot be implemented as prescribed within an outpatient setting. Rather than striving for strict manual adherence, inpatient services may focus on using the principles of FBT as a guiding framework that supports clinical coherence while remaining responsive to the structural and relational demands of inpatient care. Second, the results suggest the importance of structured and ongoing staff training when implementing or sustaining a FBT orientation in inpatient settings. Initial training alone appears insufficient; instead, continuous supervision that explicitly links theory to everyday clinical challenges is central to maintaining a shared therapeutic stance over time. Regular, model-informed supervision may also help mitigate the risk of theoretical drift, particularly in settings characterized by staff turnover and high clinical complexity. Finally, the study underscores the value of developing and sustaining a shared professional language among staff. Access to a common, theoretically anchored language, such as the “FBT language” described by participants, appears to facilitate communication, reinforce therapeutic intentionality, and support consistent clinical decision-making both within the unit and across levels of care. For inpatient services, investing in shared language development through training and reflective supervision may therefore represent a key mechanism for strengthening treatment integrity, interdisciplinary collaboration, and family engagement.

## Data Availability

The data are available from the corresponding author upon reasonable request.

## Abbreviations

AN: Anorexia nervosa
ED: Eating disorder
EDU: Eating disorder unit
FBT: Family-based therapy
TA: Thematic analysis
RTA: Reflexive thematic analysis
